# The accessory role of the outer membrane porin protein MspD in Mycobacterium smegmatis zinc homeostasis

**DOI:** 10.1099/mic.0.001699

**Published:** 2026-04-21

**Authors:** Elke Goethe, Martina Ackermann, Larissa Bulmann, Kristin Laarmann, Astrid Lewin, Janita Luehrs, Mathias Muesken, Ralph Goethe

**Affiliations:** 1 Institute of Microbiology, Department of Infectious Diseases, University of Veterinary Medicine Hannover, Hannover, Germany; 2 Unit 16 Mycotic and Parasitic Agents and Mycobacteria, Robert Koch-Institute, Berlin, Germany; 3 Central Facility for Microscopy, Helmholtz Centre for Infection Research, Braunschweig, Germany

**Keywords:** metal transport, porins, zinc, zinc homeostasis, Zinc Uptake Regulator

## Abstract

Zinc is the second most abundant transition metal in living cells and is involved in numerous biological processes. Regulation of *Mycobacterium smegmatis* (MSMEG) zinc homeostasis and zinc-specific importers ZnuABC and ZnuABC2 in the mycobacterial cytoplasmic membrane are well described; however, transport across the outer membrane (OM) remained unexplored so far. MspD is a porin highly similar to MspA, the major and well-studied porin responsible for the uptake of several compounds. Unlike MspA and the paralogues MspB and MspC, MspD was found to be transcriptionally induced upon zinc starvation and deletion of the global Zinc Uptake Regulator ZUR. Our study aimed to investigate the role of *msp*D (*msmeg*_6057) in MSMEG zinc homeostasis as well as the regulation of MspD expression. In addition, by growth experiments, quantitative real-time PCR analyses, *in situ* gene tagging and electron microscopy, we successfully demonstrated the zinc-responsive expression of the MspD protein as well as its complex formation and incorporation into the OM. Overall, the results provided strong evidence for an involvement of the MspD protein in zinc homeostasis of MSMEG.

## Data Summary

Sanger sequencing data is available at EMBL-EBI through accession no. PRJEB104607.

## Introduction

After iron, zinc is the second most abundant transition metal ion in both the environment and living organisms. It is essential for many biological processes and crucial for bacterial survival, as it is an important factor in enzymatic reactions, DNA binding and gene regulation as well as host–bacteria interaction. However, zinc bioavailability is highly variable in the environment. In hosts, it is actively altered during innate immune defence to limit bacterial growth through zinc sequestration or overload [1,2]. To cope with this, bacteria tightly regulate zinc homeostasis through specialized storage and transport systems [3,7].

Impermeable cell membranes are major obstacles for ion homeostasis of bacteria. This is further complicated in bacteria with a cell envelope consisting of two membranes such as Gram-negative bacteria and mycobacteria. The inner cytoplasmic membrane (CM) is very similar in both, consisting of a phospholipid bilayer, which in mycobacteria is associated with different lipoglycans. The CM of Gram-negatives and mycobacteria is overlayed with a thin peptidoglycan layer in the periplasmic space. However, the outer envelopes differ markedly: Gram-negative bacteria have an asymmetric outer membrane (OM) with phospholipids in the inner leaflet and LPS in the outer leaflet, whereas mycobacteria possess a unique OM formed by a covalently linked peptidoglycan–arabinogalactan core esterified with long-chain mycolic acids, creating a highly hydrophobic mycomembrane as the outermost lipid barrier [8]. This architecture sets mycobacteria apart from most other prokaryotes and confers resistance to environmental stressors while at the same time severely limiting nutrient uptake due to its extreme hydrophobicity [9,12].

These cell envelope complexities illustrate that zinc transport systems must be adapted to the structure of the bacterial cell envelope. Gram-negatives, such as *Escherichia coli*, *Yersinia ruckeri* and *Acinetobacter baumannii* [13,15], established high-affinity zinc transport systems like ZnuABC importers in the CM and TonB-dependent transporters such as ZnuD of *Neisseria meningitidis* as well as porins such as *E. coli* OmpF for passive diffusion in the OM [16,18].

In mycobacteria, such as the nonpathogenic model organism *Mycobacterium smegmatis* (MSMEG), zinc transport across the CM is mediated by ZnuABC (*msmeg*_6045–47) and ZnuABC2 (*msmeg*_6049–52) [14,16]. These transporters are essential for zinc acquisition and are regulated by the Zinc Uptake Regulator (ZUR) in a zinc-dependent manner [15,16]. Mycobacterial TonB-dependent transporters involved in zinc uptake are so far not described [19]. In the presence of zinc, ZUR binds to specific DNA operator sequences, thereby repressing the expression of zinc importer genes. Under zinc-limiting conditions, ZUR binding is relieved, permitting transcription of these genes to facilitate zinc uptake. However, zinc translocation through the mycobacterial OM is so far unexplored. In fast-growing mycobacteria, nutrient and ion transport across the OM is facilitated mostly by non-specific membrane-spanning porins that densely cover the OM. In MSMEG, the prototype protein for these porins – MspA (*msmeg*_0965) – has been identified and well-characterized [10,20,22]. It represents a self-assembling protein forming stable octameric complex with a central negatively charged pore for the passive diffusion of small molecules and cations through the OM [23,24]. MspA is the most abundant porin in the OM of MSMEG; however, additional Msp family members have been identified. While MspB (*msmeg*_0520), MspC (*msmeg*_5483) and MspD (*msmeg*_6057) constitute paralogues of MspA, MspB and MspD are thought to act as backup porins since their expression was activated upon deletion of MspA [23]. Msp porins have been implicated in the transport of sugars, amino acids, phosphate, antibiotics and even low-affinity metal uptake of iron and copper [23,25,29]. Similar porins are absent in slow-growing species such as *Mycobacterium tuberculosis* (MTB) [22,30]. Deletion of porins in MSMEG and *Mycobacterium abscessus* increased drug resistance, intracellular persistence and, in some cases, virulence [26,31,33]. In contrast, heterologous expression of *msp* genes in slow-growing species like *Mycobacterium bovis* and MTB enhanced growth and increased susceptibility to antibiotics or copper [28,34, 35], indicating their importance for environmental survival.

The presence of four Msp proteins, as well as their differential expression, suggests that each has distinct metabolic relevance. Consistent with this idea, Zhang *et al*. recently demonstrated that Msp proteins share some functions but also exhibit specific roles for the translocation of nutrients and certain metals. In particular, MspD was shown to facilitate copper and iron uptake when overexpressed in an MSMEG mutant lacking all Msp porins [29].

Recently, we found that the expression of *msp*D, but not *msp*A-C, was strongly induced under zinc starvation and in an MSMEG *zur* deletion mutant, suggesting that MspD contributes to ZUR-regulated zinc homeostasis [6]. Here, we provide evidence that MspD contributes to zinc translocation across the OM, confirming a specific role of MspD in zinc homeostasis of MSMEG.

## Methods

### Bacterial strains, standard growth conditions and chemicals

All strains used are listed in Table S1 (available in the online Supplementary Material) and plasmids and primers in Tables S1 and S2, respectively. Chemicals were purchased from Carl Roth (Karlsruhe, Germany) or Sigma-Aldrich (Munich, Germany) if not stated otherwise.

*E. coli* 10-beta was used for plasmid cloning and grown in Luria–Bertani (LB) broth (37°C/200 r.p.m.) or on LB agar plates (37°C). Competent cells were prepared according to Sambrook and Russell [36].

*M. smegmatis* mc^2^ 155 wild-type (MSwt), mutant strains MS∆*znu*ABC (MS∆1), MS∆*znu*ABC2 (MS∆2), MS∆*msp*D (MS∆3), MS∆*znu*ABC∆*znu*ABC2 (MS∆∆4), MS∆*znu*ABC∆*msp*D (MS∆∆5), MS∆*zur* (MS∆6), MS∆*znu*ABC∆*znu*ABC2∆*msp*D (MS∆∆∆7) and complemented mutant strains (Table S1) were grown in Difco 7H9 Middlebrook medium (Becton Dickinson, Franklin Lakes, NJ, USA) containing 10% OADC (0.06% Oleic acid, 5% Albumin, 2% Dextrose, 0.085% NaCl, 0.003% Catalase), 2.5% glycerol and 0.025% tyloxapol (MBXT) or in Sauton’s medium (SM) (0.05% KH_2_PO_4_, 0.2% citric acid, 0.005% FeNH_3_-citrate, 0.4% l-asparagine monohydrate, 6% glycerol, 0.05% MgSO_4_, 0.025% tyloxapol, pH 7.4) at 37°C/150 r.p.m. or on LB agar or Difco Middlebrook 7H10 agar (Becton Dickinson) containing 10% OADC and 2.5% glycerol (37°C). SM was prepared in purified Milli-Q® water in rinsed plastic ware, treated with 1% Chelex overnight, filtrated and autoclaved. Media were supplemented with kanamycin (broth: 20 µg ml^−1^, agar plate: 50 µg ml^−1^) or hygromycin (broth: 50 µg ml^−1^, agar plate: 100 µg ml^−1^) if necessary. Electrocompetent cells were prepared according to Parish and Stoker [37] or as described in section ‘Generation of *in situ* HA-tagged *msp*D strain’.

### Growth experiments, spot-on assays and time-course experiments

Growth experiments and spot-on assays with MSwt, MS∆*msp*D, MS∆*znu*ABC∆*msp*D, MS∆*znu*ABC∆*znu*ABC2∆*msp*D and complemented strains in SM were performed as described earlier [38]. In brief, bacteria were precultured in MBXT to an OD_600_ (OD 600 nm) of 1.0, harvested, washed and inoculated with an OD_600_ of 0.1 into fresh SM with or without supplementation with 10 µM ZnSO_4_. Growth was monitored daily for 8 days by OD_600_ measurement. For spot-on assays, bacteria were grown in MBXT, serially diluted in PBS and spotted on 7H10 standard agar or 7H10 supplemented with 1 or 5 µM of the zinc chelating agent *N*,*N*,*N*′,*N*′-tetrakis(2-pyridylmethyl)ethylenediamine (TPEN, Thermo Fisher Scientific, Darmstadt, Germany) or 25 or 100 µM of different single cations (ZnSO_4_, MnSO_4_ and FeNH_3_-citrate). Agar plates were incubated for 4 days at 37°C. For time-course gene expression experiments, MSwt was grown in MBXT to an OD_600_ of 1.0, treated with 10 µM TPEN and samples were taken at 0/3/6/9/12/15/18/21/24/27/30/60 min. Transcription was blocked with a stop solution containing 95% ethanol and 5% phenol, and samples were snap frozen with liquid nitrogen prior to RNA extraction. For time-dependent protein expression, please refer to section ‘Analysis of MspD protein expression’.

### Preparation of nucleic acids, cDNA synthesis and quantitative real-time PCR

Extraction of genomic DNA was performed according to Stratmann *et al*. [39]. Plasmid DNA was prepared with a NucleoSpin Plasmid extraction or NucleoBond AX kit (Macherey and Nagel GmbH, Düren, Germany) according to the manufacturer’s protocol. All plasmids were checked by sequencing (Microsynth AG, Balgach, Switzerland). Total RNA was prepared using Direct-zol RNA miniprep kit according to the manufacturer’s protocol (Zymo Research, Bath, UK), and cDNA was synthesized and analysed for expression of zinc-responsive genes *znu*A (*msmeg*_6047, primers 22/23), *znu*A2 (*msmeg_*6049, primers 20/21), *rpm*G (*msmeg_*6067, primers 22/23) and *msp*D (*msmeg*_6057, primers 9/10), as well as *msp*A (*msmeg*_0965, primers 24/25) by quantitative real-time PCR (qRT-PCR) as described earlier [40]. *gapdh* (*msmeg*_3084, primers 1/2) was used as a housekeeping gene for normalization, and data are presented from three biological replicates as ∆∆ct or ∆ct. Primer efficacies were evaluated using serial dilutions of genomic DNA.

### Generation and complementation of unmarked mutant strains

Mutant strains MS∆*msp*D, MS∆*znu*ABC∆*msp*D and MS∆*znu*ABC∆*znu*ABC2∆*msp*D (Table S1) were generated by use of the p2NIL-pGOAL system published by Parish and Stoker [41] according to the protocol by Goethe *et al*. [6]. In brief, construction of the p2NIL-manipulation plasmid p2NIL-MS6057_Del for the deletion of *msp*D in several strains 1.5 kb upstream (A) and downstream (B) of the deleted region was amplified by PCR with Q5® high-fidelity polymerase (NEB) from genomic DNA of MSwt using primers 3/4 (A) and 5/6 (B). A and B fragments were subcloned into pJet^TM^1.2, sequenced, digested with *Hind*III/*Bbs*I (A) and *Bbs*I/*Kpn*I (B) and ligated simultaneously to *Hind*III/*Kpn*I-digested p2NIL. The resulting plasmid p2NIL-MS6057_AB was digested with *Pac*I and ligated to a marker gene cassette, obtained by *Pac*I digestion from pGOAL19, resulting in deletion plasmid p2NIL-MS6057_Del. Transformation of MSwt, MS∆*znu*ABC and MS∆*znu*ABC∆*znu*ABC2 electrocompetent cells [38] with this deletion plasmid and selection of mutants was performed as described before [6], resulting in strains MS∆*msp*D, MS∆*znu*ABC∆*msp*D and MS∆*znu*ABC∆*znu*ABC2∆*msp*D, respectively. Deletion of *msp*D was confirmed by PCR with primers 7/8 and transcription control by qRT-PCR with primers 9/10.

Mutant strains were complemented homologously with *msp*D under control of its own promoter. Complementation plasmid F3 was constructed as follows: *msp*D and 483 bp upstream *msp*D were amplified by standard PCR with Q5® high-fidelity polymerase from genomic DNA of MSwt with primers 11/12, and *Xba*I/*Hind*III-digested PCR product was ligated into *Xba*I/*Hind*III-digested pMV306hyg. Plasmid F3 was transformed into MS∆*msp*D, MS∆*znu*ABC∆*msp*D and MS∆*znu*ABC∆*znu*ABC2∆*msp*D mutant strains, resulting in complemented strains MS∆*msp*D::F3 (MS∆3::F3), MS∆*znu*ABC∆*msp*D::F3 (MS∆∆5::F3) and MS∆*znu*ABC∆*znu*ABC2∆*msp*D::F3 (MS∆∆∆7::F3). Complementation was confirmed by qRT-PCR using primers 9/10 (Fig. S1).

### Generation of an *in situ* HA-tagged *msp*D strain

Plasmid pKM491 (Addgene # 109282, kindly provided by Murphy *et al*. [42]) was modified by inverse PCR with primers 13/14 to generate pKM491-HA. Presence of HA-Tag in this payload plasmid was confirmed by sequencing and transformed together with target-specific oligo oORBIT-MS_6057 HA (15, table S2) into MSwt harbouring pKM461 (Addgene # 108320, kindly provided by Murphy *et al*. [42]) to generate an *in situ* C-terminally HA-tagged *msp*D according to the protocol of Murphy *et al*. [42]. In brief, MSwt::pKM461 was grown in MBXT+20 µM kanamycin to an OD_600_ of 0.5. The expression of recombinase and annealase of pKM461 was induced upon addition of 500 ng µl^−1^ anhydrotetracycline for 2 h. After 10 min of incubation on ice, cells were harvested, washed twice with 10 ml and resuspended with 2 ml ice-cold 10% glycerol. Aliquots of 380 µl were immediately used for simultaneous electroporation (2.5 kV; 25 µF; 1,000 Ω) with 200 ng dialysed pKM491-HA and 1 µg target-specific oligo oORBIT-MS_6057 HA. Cells were resuspended in 2 ml MBXT, grown overnight at 37°C/150 r.p.m., plated on LB with 50 µg ml^−1^ hygromycin and incubated 4–5 days at 37°C. Clones were analysed for correct integration of the payload plasmid (i.e. HA-Tag) by PCR with primers 9/16 (5′-end), 17/10 (3′-end) and Western blotting.

### Analysis of MspD protein expression

For time-course expression experiments, MS::*msp*D-HA was grown in MBXT to an OD_600_ of 1.0, treated with 10 µM TPEN, and samples were taken after 0/3/6/9/12/15/18/21/24/27/30/60/120/240 min. For standard Western blotting, cells were harvested, resuspended in lysis buffer (50 mM Tris, 10 mM EDTA, 500 µM AEBSF, 150 µg ml^−1^ lysozyme, pH 7.5) and disrupted by treatment with zirconium beads in a ribolyser (four cycles, 30 s, 6 m s^−1^, intermediate cooling) and sonication (20 min, highest intensity, constant). Cell debris was removed by centrifugation (30 min/11,000 ***g***/4°C). Protein concentration was determined by MicroBCA according to the manufacturers protocol (Interchim, Montluçon, France). Fifty microgrammes of protein and 6 µl prestained protein ladder (#26619, Thermo Fisher Scientific) were loaded on a 10% SDS gel and run with buffer containing 25 mM Tris, 200 mM glycine and 0.1% SDS. Western blotting was performed on a PVDF membrane (0.45 µM pore size, Merck Millipore, Darmstadt, Germany) using SDS transfer buffer (50 mM Tris, 40 mM glycine, 20% MetOH and 0.01% SDS) for 60 min at 13 V. The membrane was blocked with 5% skim milk in Tris buffered saline containing 0.1% Tween (TBST) for 1 h at RT, incubated with anti-HA antibody (#26183, Thermo Fisher Scientific) (1:1,000 in 5% SM-TBST) overnight at 4°C and anti-mouse HRP (#7076S, Cell Signaling Technology, Cambridge, UK) (1:3,000 in 5% SM-TBST) for 1 h at RT with intermediate washing with TBST. MspD-HA was detected with HRP substrate (#34579, Thermo Fisher Scientific) in an Intas ChemoStar chemiluminescence detector. A second gel, run as described above, was stained with Coomassie blue.

For the extraction of MspD complexes, MSwt and MS::*msp*D-HA were grown as described above and treated equally with 10 µM TPEN, and samples were taken after 0/30/60 min. Cells were harvested, washed with 1× PBS and resuspended in 1 ml POP05 (100 mM Na_2_HPO_4_, 100 mM NaH_2_PO_4_, 150 mM NaCl and 0.1 mM EDTA)+0.5% OPOE (n-Octyl-oligo-oxyethylene, Santa Cruz Biotechnology, Texas, USA, #sc-286437) per 100 mg wet weight [43]. Samples were heated for 30 min at 95°C with intermediate mixing, followed by 10-min cooling on ice and centrifugation (30 min/9,000 ***g***/4°C). Supernatant was desalted using PD10 columns (Cytiva Life Sciences™TM, Wilmington, USA) and POP05 according to the manufacturer’s protocol. Protein complexes from eluates were precipitated using acetone (1:1) for 1 h on ice, followed by centrifugation (15 min/8,000 ***g***/4°C). The pellet was dried by air and resuspended in 150 µl POP05+0.5% OPOE. To analyse monomers and complexes, samples were divided and either mixed with DMSO (1:5) with subsequent heating for 30 min/95°C, acetone precipitation and centrifugation (monomers) or left untreated (complexes). Twelve microlitres of the sample and 6 µl of prestained protein ladder were loaded on a 10% SDS gel and run with a buffer containing 100 mM Tricine, 100 mM Tris and 0.1% SDS (according to Hermann Schägger [44]). Gels were either subjected to Western blotting or Coomassie staining as described above.

### Immuno-gold labelling for transmission electron microscopy

MS::*msp*D-HA was grown in MBXT to an OD_600_ of 1.0, divided and treated with 10 µM TPEN for 4 h. Bacteria were fixed in 0.1 M EM-HEPES buffer (HEPES 0.1 M, 0.09 M sucrose, 10 mM CaCl_2_, 10 mM MgCl_2_, pH 6.9) with 3% paraformaldehyde. After fixation, samples were washed with glycine (in EM-HEPES buffer) for 30 min at RT and centrifuged, and the bacterial pellet was stabilized in 2% noble agar. Agar blocks were incubated in 0.5% uranyl acetate in TE buffer (20 mM TRIS, 1 mM EDTA, pH 6.9) and dehydrated in EtOH (10%, 30% and 50% steps on ice and >50% EtOH steps at RT). Samples were subsequently infiltrated with LR White resin (RT; EtOH → 1:1, 2:1, 2×100%) and with incubation times of ~8 h, respectively, overnight. LR White samples were polymerized at 50°C for 48 h. Ultrathin sections of ~60–70 nm thickness were prepared using an Ultramicrotome Ultracut UC7 (Leica, Wetzlar, Germany) and collected on droplets of dH_2_O prior to labelling.

For labelling, the sample was incubated for 3 h at 30°C with 25 µM primary anti-HA antibody (#26183 Invitrogen, Carlsbad, USA), followed by a series of washing steps with PBS (15 s with spray bottle, three times on droplets of PBS, 5 min each). The sample was blocked with 0.1% skim milk for 5 min at RT before incubation with the secondary goat-anti-mouse IgG 10 nm gold antibody (EM.GAM10/2, British BioCell International, Cardiff, UK) 1:200 diluted in PBS supplemented with 0.5 mg ml^−1^ PEG20000 for 60 min at RT. Samples were washed for 15 s with a spray bottle containing PBS with Tween 20 and washed on 1× PBS droplet, 1× TE droplet and 1× dH_2_O droplet (5 min each). Samples were counterstained with 4% aqueous uranyl acetate for 3 min.

Images were acquired with a Libra 120 Plus (Zeiss, Jena, Germany) with an acceleration voltage of 120 kV and at calibrated magnifications. Contrast and brightness adjustments as well as size measurements were done with the ITEM software (version 5.2; Olympus Soft Imaging solutions, Münster, Germany).

To quantify the label/µm, gold particles bound to cells vs. background were counted and correlated with the according area of cells/background determined from Otsu-thresholded images using Fiji.

### Bioinformatics, statistics and data availability

Physico-chemical properties of proteins were analysed with ProtPi protein tool 2.2.29.152 (https://www.protpi.ch/). Pore models (Msp-Oktamers) have been created using protein sequences of MspA (WP_003891919.1) and MspD (WP_162139622.1) without signal sequences (File S1) with Swiss-Model Interactive Workspace [45]. Statistics for qRT-PCR were calculated from averaged duplicates of three biological replicates with GraphPad Prism 10.4.1 as described in the figure legends [46]. Data derived from Sanger sequencing are available at EMBL-EBI (accession no. PRJEB104607).

## Results

### Expression of *msp*D during zinc deficiency

**Fig. 1.**
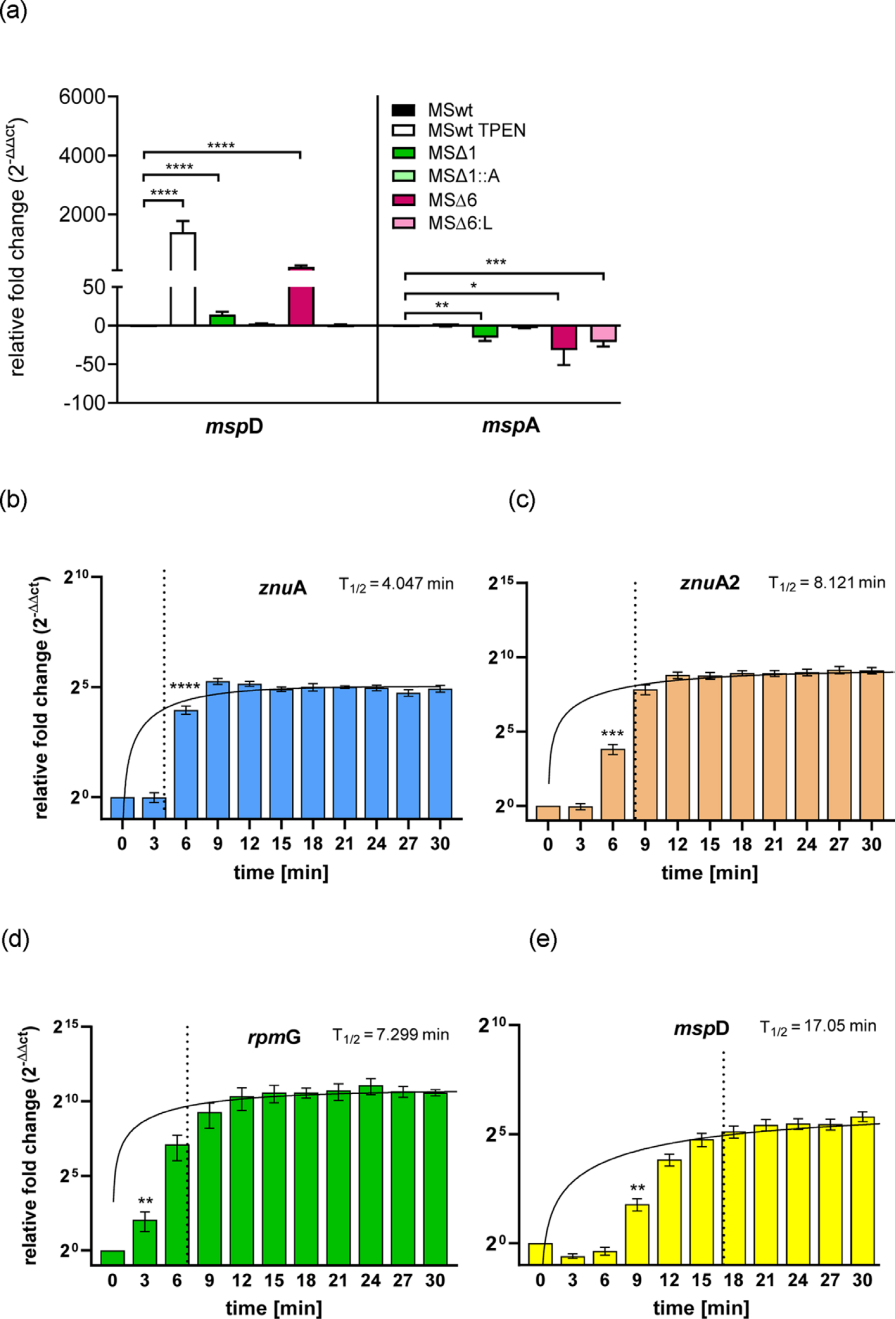
Expression of *msp*D during zinc deficiency. (**a**) MSwt, MS∆*znu*ABC (MS∆1), MS∆*zur* (MS∆6), complemented MS∆1::A and MS∆6::L strains were grown in MBXT to an OD_600_ of 1.0. MSwt was incubated with 10 µM TPEN for 2 h or left untreated. Expression of *msp*D (left, primer 9/10) and *msp*A (right, primer 24/25) in all strains was evaluated by qRT-PCR. Results are expressed as relative fold change (2^−ΔΔCt) normalized to the housekeeping gene *gap*DH and compared to the MSwt control (mean±SEM). *N*=3 in duplicate. Statistical analysis of ∆Ct values was performed using ordinary one-way ANOVA (post hoc Dunnett’s multiple comparison test) with *P≤0.05; **P≤0.005; ***P≤0.0005; ****P≤0.0001. (**b–e**) Time-dependent induction of MSMEG *msp*D and other zinc-responsive systems. MSwt was grown in MBXT to an OD_600_ of 1.0 and induced with 10 µM TPEN. Samples were taken at T0 and indicated time points. Gene expression of *znu*A (primer 18/19), *znu*A2 (primer 20/21), *rpm*G (primer 22/23) and *msp*D was determined by qRT-PCR. Results are expressed as relative fold change (2^−ΔΔCt) normalized to the housekeeping gene *gap*DH and compared to MSwt control at T0 (mean±sem). *N*=3 in duplicate. Statistical significance of gene induction was calculated from ∆Ct values with Repeated Measures (RM) one-way ANOVA (post hoc Dunnett’s multiple comparison test, *P≤0.05; **P≤0.005; ****P≤0.0001). Relative gene expression over time was fit using a one-phase exponential association model (black line). T_1/2_, defined as the time required to reach 50% of the maximal expression, was calculated from the best-fit rate constant (dashed line).

We previously demonstrated that in times of MSMEG zinc starvation, expression of the main zinc importers *znu*ABC and *znu*ABC2 and also of the porin *msp*D was induced [6,38]. Since the well-described and closely related porin MspA is located in the OM, these findings suggested that MspD might support zinc transport across the OM when zinc demand is high. To emphasize the specific response of *msp*D to zinc starvation in comparison to other porins such as *msp*A, we analysed gene expression of *msp*D and *msp*A in three zinc starvation models: a *znu*ABC deletion mutant, a *zur* mutant of MSMEG and MSMEG wild-type (MSwt) grown at standard conditions or treated with the zinc chelating agent TPEN (10 µM, 2 h), mimicking zinc starvation. The MS∆*znu*ABC mutant was used, as it exhibits a zinc starvation phenotype, characterized by an induced expression of the alternative zinc uptake system *znu*ABC2 in a previous study [38]. MS∆*zur*, which is lacking the global zinc uptake regulator ZUR, expresses all zinc uptake systems repressed by ZUR [6]. As the gene sequences of *msp*D and *msp*A are highly similar, oligonucleotide primers for PCR are located within dissimilar regions ([23], Table S2). As shown in Fig. 1(a), the expression of *msp*D was increased in MSwt treated with TPEN, MS∆*znu*ABC (MS∆1) and MS∆*zur* (MS∆6), and this effect was reversed upon homologous complementation (MS∆1::A, MS∆6::L) (Fig. 1a, left side). Induction of *msp*D in three different zinc starvation models strongly supports its zinc-dependent transcription. In contrast, *msp*A expression was not induced or reduced in mutant strains (Fig. 1a, right side) and TPEN-treated samples, suggesting a reduced expression of *msp*A under zinc-limiting conditions.

Zinc homeostasis is tightly regulated and must be continuously adapted to the increasing demand of the augmenting biomass during bacterial growth, changing environment or within the host. To cope with this, the regulation of zinc uptake systems is fine-tuned by sensitive regulators like ZUR. There is evidence that zinc-responsive systems are induced sequentially, as shown for *Bacillus subtilis* [47]. Assuming a relevance of MspD for OM zinc translocation, reduced expression of MspA suggested that MspD is getting more important when zinc starvation is developing. To investigate this, MSwt was grown to the exponential phase in standard MBXT medium and treated with 10 µM TPEN followed by time-course sampling at 3-min intervals for 30 min and after 60 min. Gene expression profiles of *znu*A (*msmeg*_6047), *znu*A2 (*msmeg*_6049), *rpm*G (*msmeg*_6067) and *msp*D at all time points were analysed (60 min not shown). We included *rpm*G in our analysis, as this and other alternative ribosomal proteins (ARPs) are known key players in mycobacterial zinc homeostasis [48,49]. A sequential pattern of gene induction could be observed (Fig. 1b–e). *rpm*G was significantly induced compared to T0 after 3 min, *znu*A and *znu*A2 after 6 min, while significant induction of *msp*D was observed after 9 min. Moreover, we calculated the time to reach 50% of maximal expression of each gene (T_1/2_), by a one-phase exponential association curve-fit model. The resulting T_1/2_ values are 4.0 min (*znu*A), 8.1 min *(znu*A2), 7.3 min (*rpm*G) and 17.1 min (*msp*D). These values indicate that MSMEG immediately produces ARP to maintain zinc-independent translation and restores zinc homeostasis by the activation of zinc uptake through ZnuABC and ZnuABC2. The delay of *msp*D is modest and occurs within a short time frame. Still, this scenario is consistent with our previous findings that ZnuABC is the main zinc importer, which is supported by ZnuABC2 in MSMEG [38]. The data also suggest that zinc translocation through the OM might initially be compensated by the standard set of OM porins and is subsequently supported by MspD.

### Only the deletion of *msp*D in combination with *znu*ABC transporters has severe effects on MSMEG growth

**Fig. 2.**
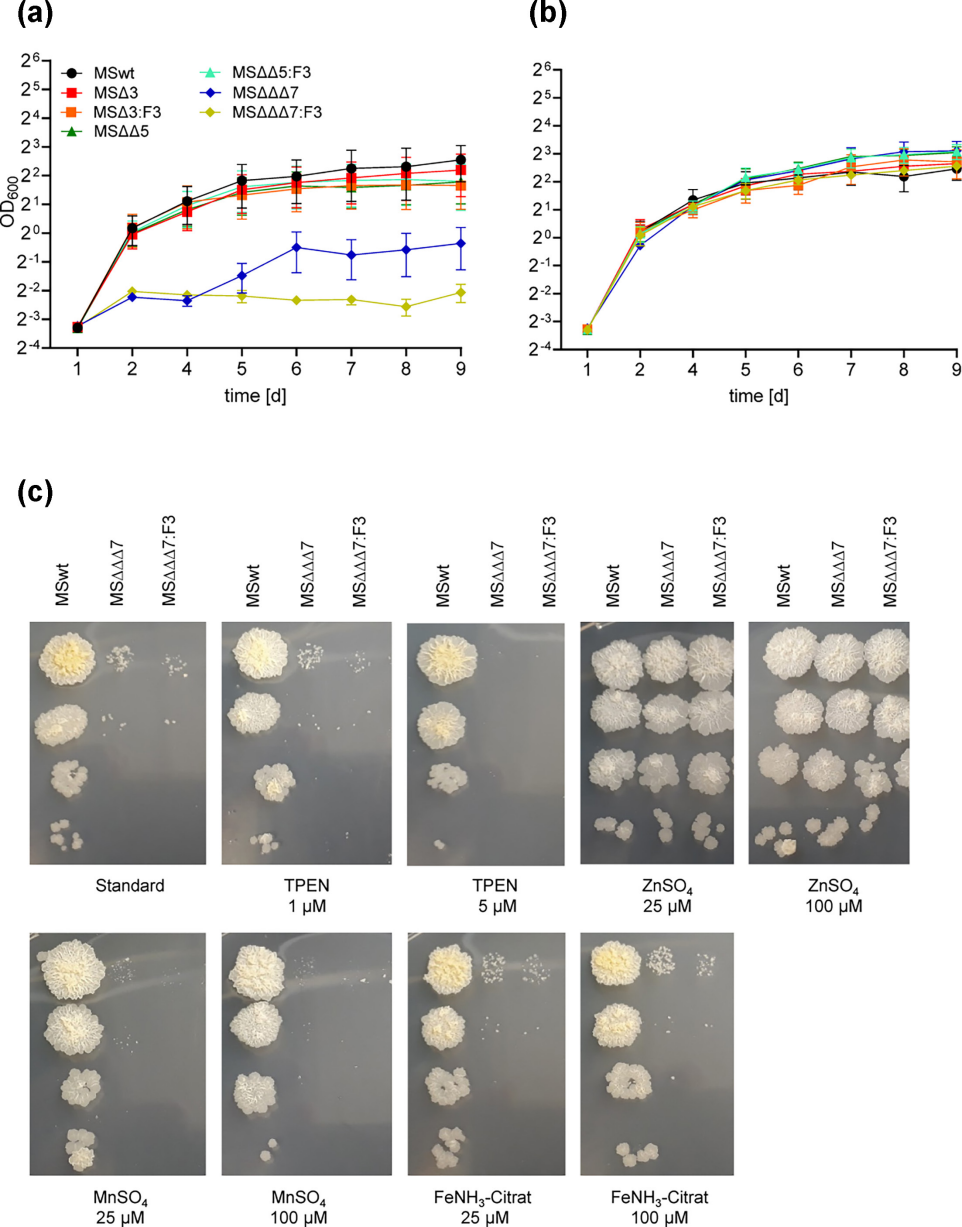
Accessory role of MspD in zinc homeostasis. MSwt, MS∆*msp*D (MS∆3), MS*∆znu*ABC*∆msp*D (MS∆∆5), MS*∆znu*ABC*∆znu*ABC*2∆msp*D (MS∆∆∆7) and *msp*D complemented strains (::F3) were grown in MBXT to an OD_600_ of 2–3, harvested, washed and inoculated to an OD_600_ of 0.1 in SM (**a**) or SM supplemented with 10 µM ZnSO_4_ (**b**). Growth was monitored daily by measurement of OD_600_ for 8 days. Shown are the results of four independent replicates in duplicate. (**c**) Spot-on assay. Strains were grown in MBXT to an OD_600_ of 2–3, harvested, washed, inoculated in 1× PBS to an OD_600_ of 0.1, serially diluted up to 10^−4^ and spotted on 7H10 standard agar or on 7H10 supplemented with indicated substances. Plates were incubated for 4 days at 37°C. Shown are representative pictures of three independent experiments.

To dissect the role of MspD for MSMEG zinc metabolism, we constructed mutant strains of either *msp*D alone (MS∆*msp*D) or in combination with *znu*ABC (MS∆*znu*ABC∆*msp*D) and *znu*ABC+*znu*ABC2 (MS∆*znu*ABC∆*znu*ABC2∆*msp*D) and complemented strains (::F3) and performed growth experiments in minimal SM. Control strains with single *znu*ABC or *znu*ABC2 deletions (MS∆*znu*ABC and MS∆*znu*ABC2, respectively [38]) were also analysed. No growth defect was visible for MS∆*msp*D (MSΔ3) and MS∆*znu*ABC∆*msp*D (MSΔΔ5) (Fig. 2a) or MS∆*znu*ABC and MS∆*znu*ABC2 (Fig. S2) and their complemented strains; all strains showed growth similar to MSwt. Contrary to this, growth of the triple mutant MS∆*znu*ABC∆*znu*ABC2∆*msp*D (MSΔΔΔ7) was severely reduced, which was only rescued upon supplementation with zinc (Fig. 2a, b) but not with other metals, as shown in a spot-on assay (Fig. 2c). This phenotype is similar to the phenotype of an earlier published double mutant lacking *znu*ABC and *znu*ABC2 (MSΔΔ4), which also showed hampered growth in SM, which could be restored by zinc supplementation [38]. Complementation of MS∆*znu*ABC∆*znu*ABC2∆*msp*D with *msp*D alone was however not sufficient to restore the growth defect (Fig. 2a, c), despite its up-regulation upon zinc starvation. This discrepancy is likely attributed to the severity of the zinc starvation phenotype of this mutant.

### Relevance of *mspD* for zinc import indicated by altered RNA expression of zinc marker genes in *mspD* deletion mutants

We then aimed to assess the relevance of *msp*D in zinc uptake by examining its impact on transcriptional regulation in *msp*D mutant strains. This method offers a more sensitive measure of alterations in zinc homeostasis as compared to growth-based assays. To do this, we quantified expression levels of the remaining zinc uptake systems *znu*ABC (represented by *znu*A), *znu*ABC2 (represented by *znu*A2) and *rpm*G in the different *msp*D mutant strains. As controls, and to distinguish specific expression effects, we also analysed gene expression in *znu*ABC/*znu*ABC2 single or double mutants and *msp*D complemented strains (see below).

**Fig. 3.**
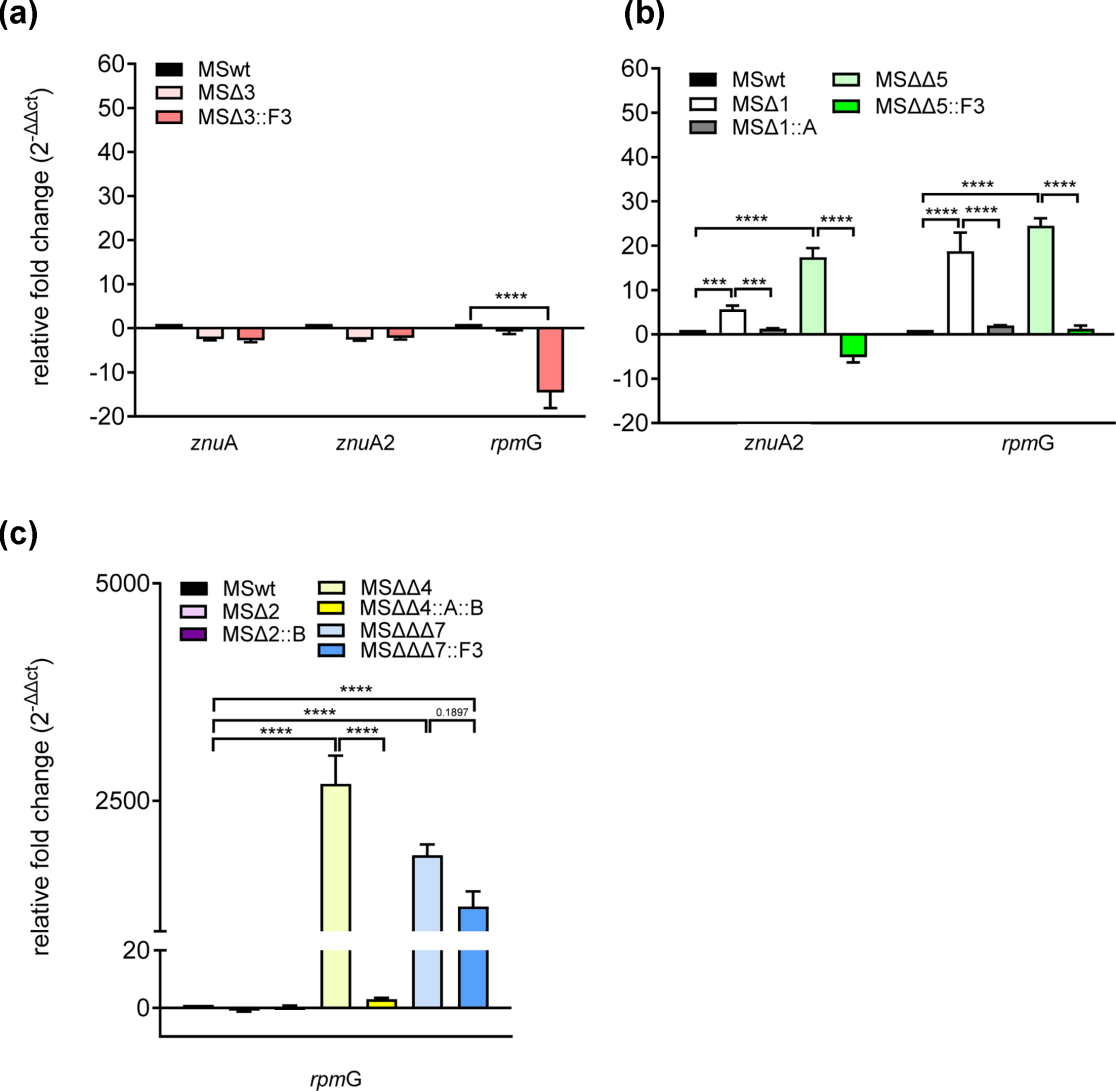
MspD contributes to zinc homeostasis. Analysis of compensatory expression of remaining zinc uptake systems in *msp*D mutant strains. All strains were grown in MBXT to an OD_600_ of 1.0. Indicated MSMEG strains were then treated with 10 µM TPEN for 2 h or left untreated. Gene expression was analysed by qRT-PCR. (**a**) Expression of *znu*A, *znu*A2 and *rpm*G in MS∆*msp*D (MS∆3) and complemented MS∆3::F3. (**b**) Expression of *znu*A2 and *rpm*G in MS∆*znu*ABC∆*msp*D (MS∆∆5), MS∆1 and complemented strains. (**c**) Expression of *rpm*G in MS∆*znu*ABC∆*znu*ABC2∆*msp*D (MS∆∆∆7), MS∆*znu*ABC∆*znu*ABC2 (MS∆∆4), MS∆2 and complemented strains. Results are expressed as relative fold change normalized to the housekeeping gene *gap*DH and compared to the MSwt control (mean±sem). *N*=3 in duplicate. Statistical analysis of ∆ct values was performed using ordinary one-way ANOVA (post hoc Šídák’s multiple comparisons test) with ***P≤0.0005 and ****P≤0.0001.

Apart from the reduced expression of *rpm*G in the complemented strain MSΔ*msp*D::F3 (MSΔ3::F3), no significant changes in gene expression were observed in the MSΔ*msp*D (MSΔ3) mutant, indicating that deletion of *msp*D alone does not markedly alter zinc homeostasis (Fig. 3a). In contrast, the double mutant MSΔ*znu*ABCΔ*msp*D (MSΔΔ5) showed significant induction of *znu*B2 and *rpm*G. These effects were also observed in the *znu*ABC single mutant MSΔ1 (main zinc importer). Notably, the elevated expression levels in MSΔ*znu*ABCΔ*msp*D (MSΔΔ5) were significantly reduced upon complementation with *msp*D alone, indicating a direct influence of *msp*D on zinc homeostasis (Fig. 3b). The expression of *znu*A2 and *rpm*G in MSΔ*znu*ABC (MSΔ1) complemented with *znu*ABC alone was similarly reduced. We did not compare expression levels between different mutant strains, as ectopic complementation can lead to different expression levels in the complemented mutant strains, depending on the site of plasmid insertion.

In the triple mutant MS∆*znu*ABC∆*znu*ABC2∆*msp*D (MSΔΔΔ7) *rpm*G, the expression was highly induced, similar to what was observed in MS∆*znu*ABC∆*znu*ABC2 (MSΔΔ4) (Fig. 3c), indicating severe zinc starvation in both mutants. Deletion of *znu*ABC2 alone (MS∆2) had no effect (Fig. 3c). Full complementation of MS∆*znu*ABC∆*znu*ABC2 (MSΔΔ4) with both *znu*ABC transporters significantly reduced *rpm*G expression to wild-type levels. Complementation of MS∆*znu*ABC∆*znu*ABC2∆*msp*D (MSΔΔΔ7) with *msp*D alone had a slight but not significant effect (*P*=0.1897), further supporting the severe character of zinc starvation in a mutant lacking all known zinc uptake systems. Overall, these analyses further indicate a contribution of MspD to zinc homeostasis.

### Zinc-regulated expression of MspD in the mycobacterial OM *in vivo*

**Fig. 4.**
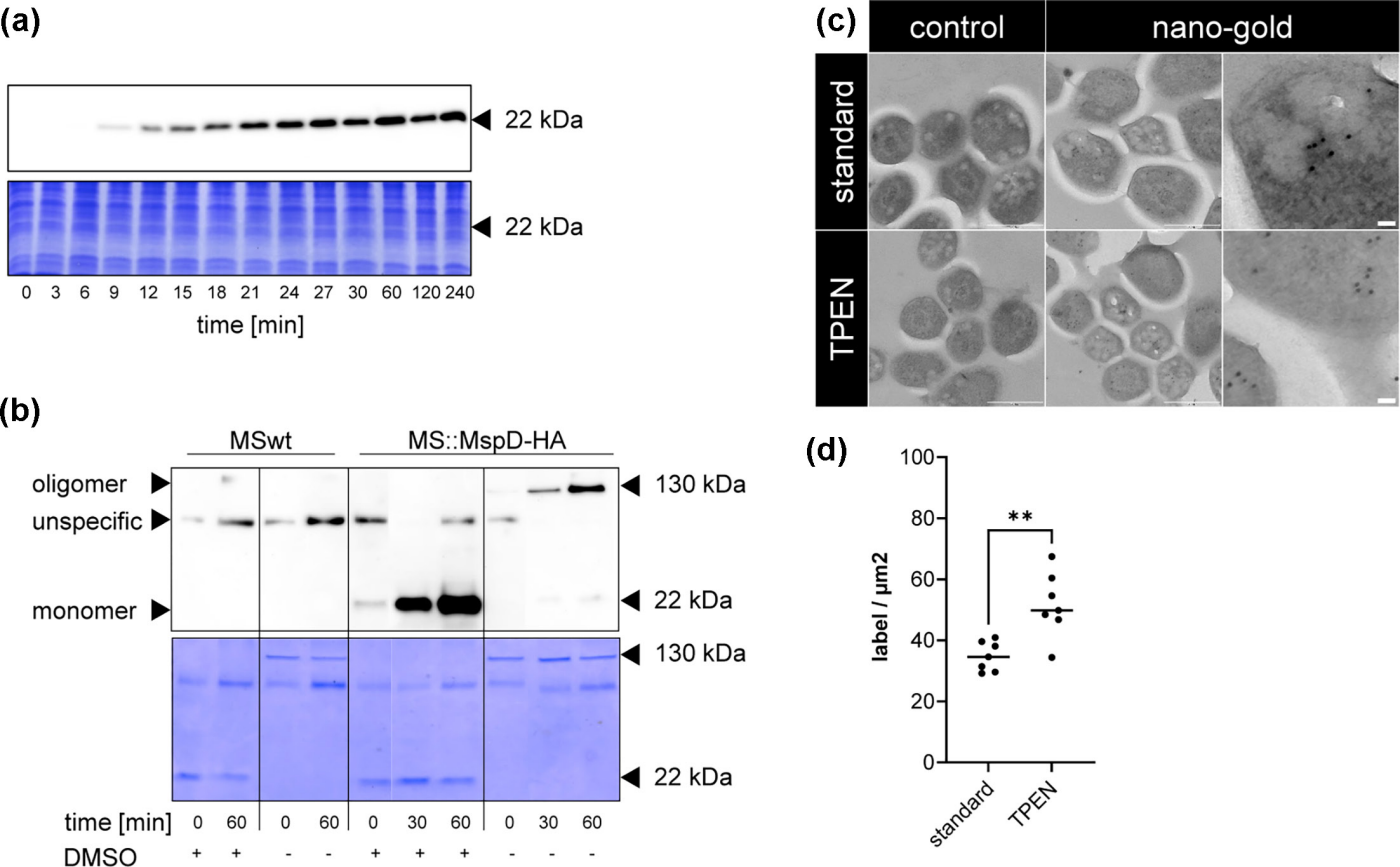
MspD expression and complex formation. (**a**) MS::MspD-HA strain with *in situ* HA-tagged *msp*D was grown in MBXT to an OD_600_ of 1.0 and treated with 10 µM TPEN for up to 4 h. Samples were taken at indicated time points. Whole cell lysates were prepared and same amounts (50 µg) were subjected to SDS-PAGE (10%) with subsequent Western blotting (upper panel) or Coomassie staining (lower panel). (**b**) MSwt and MS::MspD-HA were grown as described in (**a**) and treated with 10 µM TPEN for indicated time points. POP05+OPOE extracts were then treated either with 37°C (-) or 95°C/DMSO (+) and subjected to SDS-PAGE (10%) and Western blotting (upper panel) or Coomassie staining (lower panel). (**c**) MS::MspD-HA was grown as described above and treated with 10 µM TPEN for 4 h. Cells were fixed and incubated with anti-HA antibody and 10 nm nanogold. Control was treated with nanogold only. Scale bars equal 500 nm (left/middle images) or 50 nm (right images). Original size images are available in the supplemental data. (**d**) Nanogold labels of seven images of MS::MspD-HA standard or TPEN samples were counted and calculated as label/µm^2^. Statistical analysis was performed using unpaired t-test with **P≤0.005.

MspD peptides have been detected by mass spectrometry by Fishbein *et al*. in 2021 [50], indicating MspD expression in MSMEG. However, the detection of full-length proteins or complexes was missing to date. The above data suggested a role of MspD in zinc uptake. Therefore, we were interested in MspD protein expression and presence in the OM. To overcome misdetection due to the high structural similarity of Msp porins, we constructed a *msp*D strain with a C-terminally HA-tag inserted *in situ* using ORBIT (oligonucleotide-mediated recombineering followed by Bxb1 integrase targeting). This approach employs an *att*B site carrying payload plasmid to mediate tagging, which is co-transformed with a target-specific, *att*P site containing oligonucleotide, into an MSMEG strain expressing RecT annealase and Bxb1 integrase [42]. This allows precise, site-directed genomic modification. The resulting strain MS::*msp*D^HA^ was then grown either in the presence or absence of TPEN, following the time scheme of gene expression analysis (Fig. 1b–e). With this approach, we could confirm protein expression and zinc starvation-dependent induction of MspD by SDS PAGE and Western blotting, as shown in Fig. 4(a). This observation is consistent with our qRT-PCR data, demonstrating simultaneous gene expression and protein production starting after 9-min TPEN treatment (Fig. 1e). However, in whole cell lysates, we could only detect MspD monomers. Therefore, to enrich OM porin proteins, a selective extraction method for OM-localized MspA developed by Heinz and Niederweis [43] was applied to better analyse MspD complex formation in the OM. This method is based on boiling the samples in a phosphate-based extraction buffer (POP05) containing the detergent n-Octyl-oligo-oxyethylene (OPOE), thereby extracting OM proteins [43]. To do so, MSwt and MS::MspD-HA were grown with TPEN for 30 or 60 min, or left untreated (0, control). Proteins were extracted, divided and either incubated at 37°C (to analyse complexes) or treated with DMSO (to detect monomers) [21]. These samples were analysed by Tricine-SDS-PAGE, Western blotting and Coomassie staining. In the Western blot, protein complexes with a size of ~130 kDa could be detected in extracts of MS::MspD-HA without DMSO treatment (Fig. 4b). In DMSO-treated MS::MspD-HA extracts, the complexes could be visualized as monomers with a size of ~22 kDa. Neither MspD complexes nor monomers could be detected in samples of MSwt after Western blotting, despite successful extraction of Msp porins visualized as protein bands of 130 and 20 kDa, respectively, by Coomassie staining (Fig. 4b) . Interestingly, the amount of complexes and monomers in the Coomassie gel was similar in all samples of MSwt and MS::MspD-HA, while Western blotting showed an increase of MspD fractions over time. This indicated that the POP5-OPOE method extracts different Msp proteins including increasing amounts of MspD. Together, these experiments indicate an oligomeric organization of MspD, similar to what was observed for MspA porins, in the OM of MSMEG [51]. To support this, the expression and accumulation of MspD upon TPEN treatment in cells was analysed by transmission electron microscopy. As shown in Fig. 4(c), nanogold-labelled proteins could be visualized in both samples, however, to a significantly higher extent in samples treated with TPEN (Fig. 4d). Overall, these experiments for the first time show the zinc-dependent expression of MspD proteins, the formation of MspD complexes and *in vivo* accumulation of MspD upon zinc starvation in the OM.

### Properties of MspD pores

There is a general consensus that the translocation of substrates as well as bivalent cations through the OM in MSMEG and other fast-growing mycobacteria is mediated by porins formed by the Msp proteins A-D. MspA-D proteins show extremely high similarities, differing in a maximum of 18 amino acids between the most divergent MspA and MspD. However, they are definitely involved in the translocation of different substrates [29]. Zhang *et al*. reported that MspD is more specialized than MspA and MspC, showing a preference for cations such as copper and iron. The detection of MspD complexes in the OM, together with its zinc-dependent expression, prompted us to investigate its complex formation in more detail, to obtain evidence supporting its specificity in zinc translocation – or cation uptake in general. We therefore compared properties of the most divergent proteins MspA and MspD by *in silico* analysis.

**Fig. 5.**
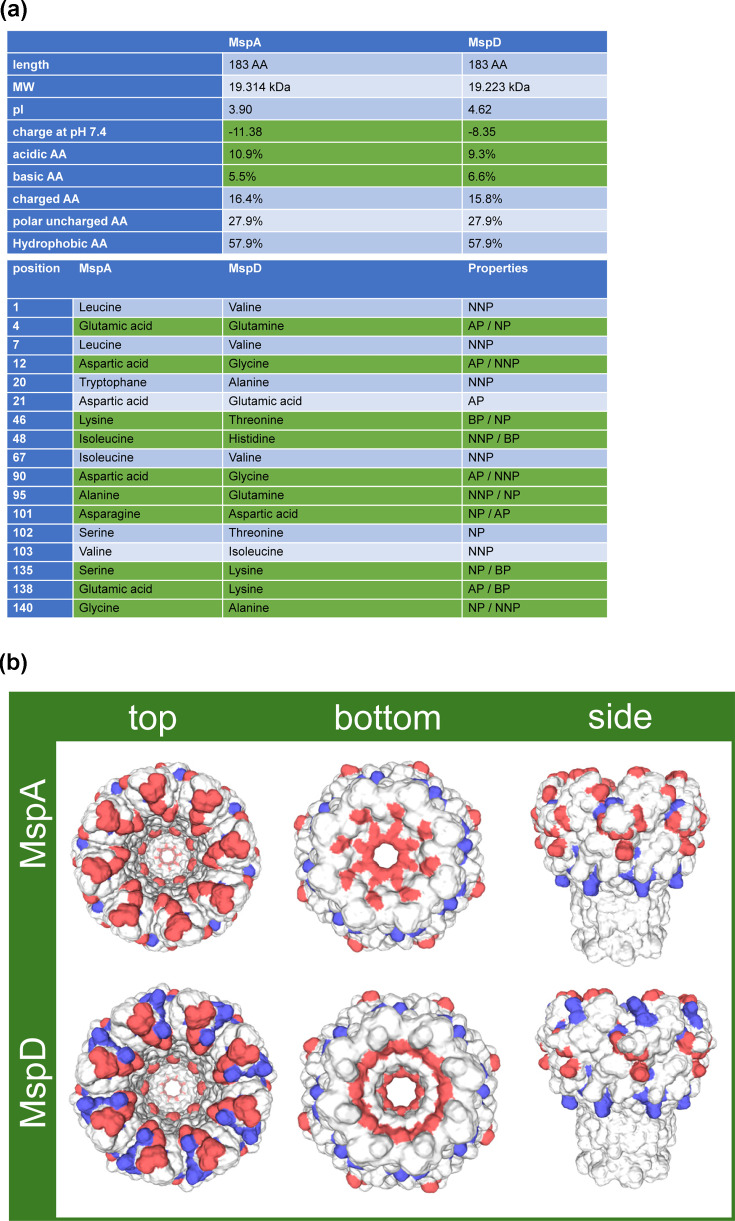
(**a**) Comparison of molecular and physico-chemical properties of MspA and MspD (without signal sequence) as well as listing of diverging amino acids and their properties using ProtPi tool. Diverging amino acids and properties are highlighted in green. NNP=neutral, non-polar; AP=acidic, polar; NP=neutral, polar; BP=basic, polar. (**b**) Models of Msp porins. Protein sequences of MspA and MspD excluding the signal sequence have been subjected to Swiss-Model Interactive Workspace [45] to form homooctamers. The models have been set to charged surface, highlighting charge differences. Red indicates acidic amino acids, blue basic amino acids.

As shown in Fig. 5(a), the net charges of MspA and MspD proteins differ significantly: −11.38 and −8.35, respectively. These differences are caused by variations in the polarity of 10 out of the 18 diverging amino acids (Fig. 5a, green, one amino acid is absent in MspD). Structural models of MspA and MspD octamers (Fig. 5b) reveal an accumulation of basic amino acids at the top of the MspD pore and a less pronounced constriction zone at the base. In MspA, B and C, this constriction zone is defined by the carboxylates of Asp^90^ and Asp^91^, which reduce the permeability for polar solutes. Notably, MspD contains a neutral glycine at position 91, reducing the local charge, potentially the reason for widening the pore and allowing less selective translocation of polar nutrients including zinc [20].

In conclusion, transcriptional, translational and *in silico* analyses give strong evidence for the role of MspD in zinc homeostasis.

## Discussion

Zinc plays a critical role in bacterial growth, survival and pathogenicity of mycobacteria [40,52, 53]. However, the highly hydrophobic mycobacterial OM poses a significant challenge for the acquisition of nutrients such as zinc. While zinc translocation through the CM of mycobacteria is mediated by specific zinc importers such as ZnuABC and is well described [38,54], translocation of zinc across the OM has not yet been characterized. In other bacteria, TonB-dependent transporters are involved in this process. To date, only one TonB-dependent transporter (FecA) was described in mycobacteria (MTB); however, this is related to uptake of iron siderophores, and to our knowledge, no TonB-dependent transporter is known for zinc uptake [19]. Here, we provided strong evidence that the OM porin protein MspD is involved in zinc homeostasis in MSMEG.

Enhanced *msp*D expression upon zinc starvation and deletion of the global zinc uptake regulator ZUR strongly supported an involvement of MspD in zinc homeostasis of MSMEG. To better assign its relevance, we firstly analysed the time-point of induction of *msp*D in comparison to *znu*ABC, the supporting *znu*ABC2 and the alternative ribosomal (ARP) *rpm*G, since it is known that ZUR targets respond hierarchically to zinc depletion [47,55]. We observed an early induction of *rpm*G (3 min), followed by the *znu*ABC transporters (6 min), which was comparable with *B. subtilis* [47]. *msp*D induction was the latest (9 min). This is congruent with the finding that ZUR binds with the highest affinity to ARP promoters, followed by *znu* and lower-affinity promoters and could also apply to our target genes. The affinity of promoters often follows biological relevance, i.e. the immediate and strong mobilization of intracellular zinc pools by the employment of ARPs followed by zinc import via high-affinity *znu*ABC transporters in several bacterial species [6,47, 55]. The slightly retarded induction of *msp*D might therefore be attributed, e.g. to a different promoter affinity or a different promoter architecture, which is reflected by the lack of a ribosome binding site and an insertion element in the 5′ untranslated region [22,23]. However, this has not yet been analysed in detail and remains to be elucidated.

We further characterized different MSMEG mutants lacking *msp*D or/and other zinc importers. Individual mutation of *msp*D did not substantially influence zinc homeostasis. Only the significantly lower expression of *rpm*G in the complemented mutant suggested that the expression of MspD in the presence of ZnuABC transporters helps to increase cellular zinc levels, thereby reducing the expression of the high-affinity ARP response system. However, more convincing evidence was provided by the deletion of *msp*D in combination with the major zinc importer *znu*ABC. This led to an increase in zinc requirement of the bacterial cell, indicated by the induction of zinc starvation responsive genes *znu*A2 and *rpm*G. Conversely, deletion of *znu*ABC alone led to increased *msp*D expression. Together, the results clearly indicated that MspD plays a role in zinc homeostasis and led to the obvious assumption that it is responsible for zinc translocation through the OM. Accordingly, by Western blotting and electron microscopy, we were able to identify zinc-inducible MspD monomers and complexes in the MSMEG OM. The size of the predicted MspD octameric complex appeared to be ~130 kDa and was, however, below what was calculated (176 kDa). Membrane *β*-barrel proteins such as Msp often bind SDS differently and migrate anomalously relative to protein markers. That can shift full octamers to ‘wrong’ apparent masses [56]. A similar phenomenon was observed for MspA [43], which is known to form a stable octameric, polar channel complex. Due to the structural similarity of MspD and MspA, most likely MspD also forms octamer homomeric porins or contributes to heteromeric Msp porins [57].

**Fig. 6.**
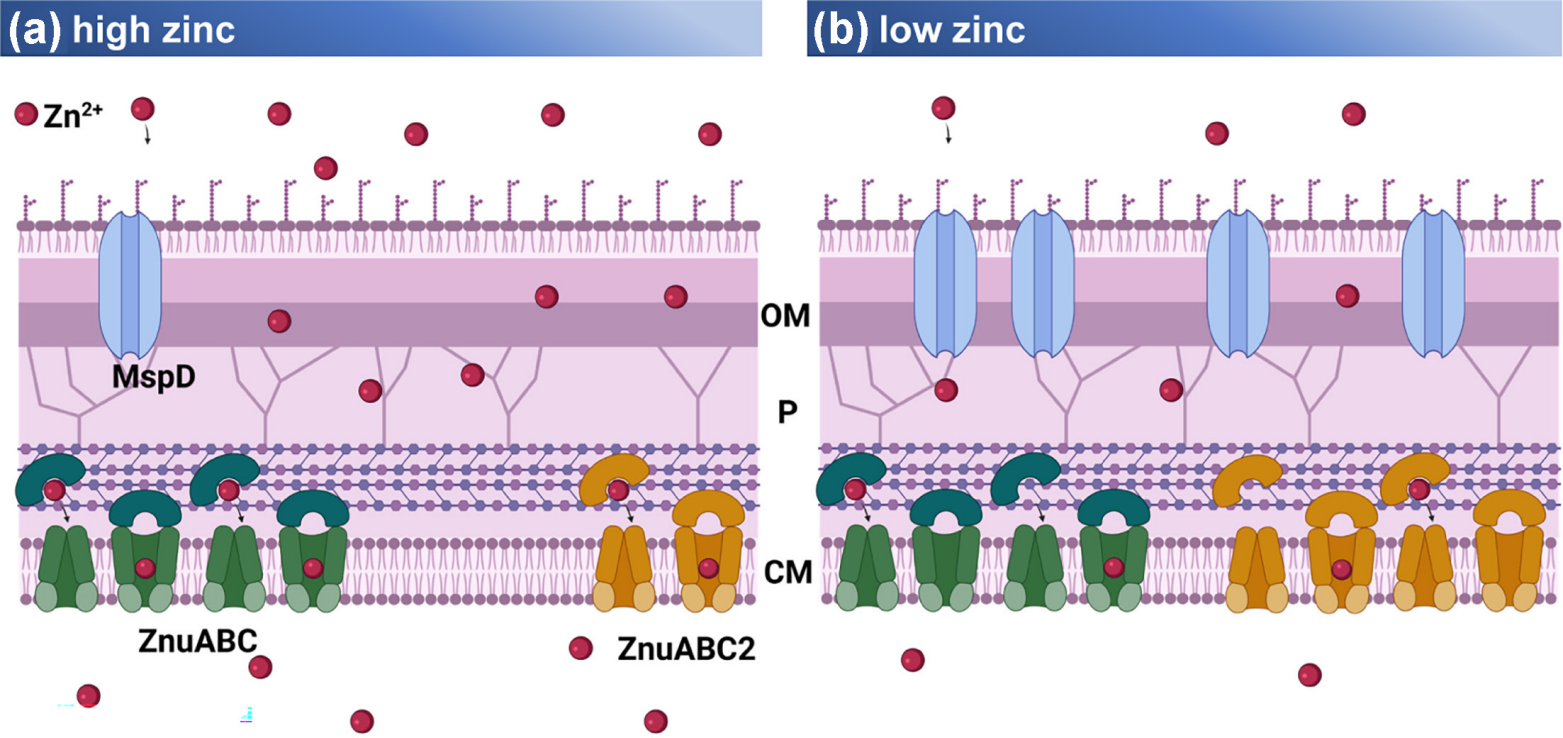
Model of zinc translocation across the mycobacterial membrane. (**a**) When zinc (red) is available, ions passively cross the OM via the porin MspD (blue) and maybe other Msp porins. In the periplasm (P), zinc ions are captured by the soluble component ZnuA (green) of the ABC-transporter ZnuABC and translocated across the CM via ZnuBC. During this state, ZnuABC is the predominant zinc importer in the CM. (**b**) When zinc is low, *msp*D expression is induced and passive translocation across the OM is facilitated by a higher number of MspD porins. Moreover, the expression of the backup transporter ZnuABC2 (yellow) in the CM is induced [38], thereby increasing the transport of zinc over the CM (created with BioRender, https://BioRender.com/g29dy1n).

The structural similarity of Msp proteins raises the question of how clear differences in substrate preferences can be explained [23,25,28]. Zhang *et al*. recently suggested that MspD shows preferences to metal ions such as copper and iron [29]. Our structural *in silico* analyses show that most plausibly better zinc translocation by MspD is due to particular structural differences to MspA, as shown in Fig. 5. Comparing MspD with MspA, only 18 amino acids are different. These amino acid exchanges reduce, however, the polarity of MspD porins which might modify substrate affinities under varying physiological conditions [57], such as zinc starvation. In agreement, Wang *et al*. [58] reported that the coordination of single metal ions in MspA channel mutants was significantly altered when amino acids Asp^90^, Asp^91^ and other sites were mutated. This holds true for MspD, which contains a neutral glycine in position 91. Similar findings were suggested for the channel protein ZntB in *E. coli*, where selective zinc uptake is attributed to a larger pore size as compared to, e.g. CorA, based on substitution of a single amino acid in the signature motif [59]. Based on all findings, we conclude that under zinc-limited conditions, the increased MspD expression contributes to higher zinc uptake by modifying the porin composition of the OM. Higher expression of MspD would permit less selective substrate transition through the OM. By this, MSMEG could also increase translocation of zinc, which further is selectively transported into the cell via specific zinc transporters ZnuABC and ZnuABC2. Interestingly, similar zinc uptake strategies have been observed in corynebacteria, such as *Synechococcus* sp., which utilize an OM porin in combination with a ZnuABC transporter in the inner membrane for zinc import [60]. We therefore propose an adapted model of zinc transporter across the OM of MSMEG, as illustrated in Fig. 6. Msp homologues are missing in pathogenic mycobacteria such as *M. bovis* and MTB, and this is related to their slow-growth phenotype [34]. Still, they do possess other types of porins, e.g. OmpA-like proteins such as OmpATb or other channel-forming proteins, which are however less efficient [61,63]. Whether and how these porins are involved in the uptake of transition metals or if slow-growing mycobacteria possess different ways of zinc uptake across the OM remains to be elucidated.

In conclusion, we are convinced that MspD contributes to increased zinc uptake across the OM during zinc starvation, reflected by transcriptional and translational changing patterns as well as structural properties.

## Supplementary material

10.1099/mic.0.001699 Uncited Supplementary Material 1.

## Abbreviations

ARP: alternative ribosomal protein
CM: cytoplasmic membrane
LB: Luria–Bertani
MSMEG: *Mycobacterium smegmatis*
MTB: *Mycobacterium tuberculosis*
OM: outer membrane
qRT-PCR: quantitative real-time PCR
SM: Sauton’s medium
TPEN: *N*,*N*,*N*′,*N*′-tetrakis(2-pyridylmethyl)ethylenediamine
ZUR: Zinc Uptake Regulator
