# Supplementary material for: The accessory role of the outer membrane porin protein MspD in Mycobacterium smegmatis zinc homeostasis
Source: Microbiology (Reading). 2026 Apr 21;172(4):001699. doi: 10.1099/mic.0.001699 (PMC13098993; doi:10.1099/mic.0.001699)
Supplement: Uncited Supplementary Material 1. [file mic-172-01699-s001.pdf]

Table T1: Bacterial strains and plasmids used in this study

Bacterial strains

Abbreviation	Strain	Description	Origin
E. coli	*Escherichia coli* 10-beta NEB(R)	Δ(ara-leu) 7697 araD139 fhuA ΔlacX74 galK16 galE15 e14- Φ80dlacZΔM15 recA1 relA1 endA1 nupG rpsL (StrR) rph spoT1 Δ(mrr-hsdRMS-mcrBC)	New England Biolabs
MSwt	*M. smegmatis* mc^2155	Transformable strain of the isolate M.smegmatis ATCC 607	Snapper et al., 1990
MSΔ1	*M* SMEGΔ*znu* ABC	MSwt transformed with p2NIL-MS6045-47_Del	Goethe et al. 2021
MSΔ2	MSMEGΔ*znu* ABC2	MSwt transformed with p2NIL-MS6049-51_Del	Goethe et al. 2021
MSΔ3	MSMEGΔ*msp* D	MSwt transformed with p2NIL-MS6057_Del	this work
MSΔΔ4	MSMEGΔ*znu* ABCΔ*znu* ABC2	MSΔ1 transformed with p2NIL-MS6049-51_Del	Goethe et al. 2021
MSΔΔ5	MSMEGΔ*znu* ABCΔ*msp* D	MSΔ1 transformed with p2NIL-MS6057_Del	this work
MSΔ6	MSMEGΔ*zur*	MSwt transformed with p2NIL-MS4487_Del	Goethe et al. 2020
MSΔΔΔ7	MSMEGΔ*znu* ABCΔ*znu* ABC2Δ*msp* D	MSΔΔ4 transformed with p2NIL-MS6057_Del	this work
MSΔ1::A	MSΔ*znu* ABC complemented with *znu* ABC	MSΔ1 complemented with pMV306kan, containing genes *msmeg_* 6045-47	Goethe et al. 2021
MSΔ2::B	MSΔ*znu* ABC2 complemented with *znu* ABC2	MSΔ2 complemented with pMV306hyg, containing genes *msmeg* _6049-51	Goethe et al. 2021
MSΔ3::F_3	MSΔ*msp* D complemented with *msp* D and own promoter	MSΔ3 complemented with pMV306hyg, containing *msmeg* _6057	this work
MSΔΔ4::A::B	MSMEGΔ*znu* ABCΔ*znu* ABC2 complemented with *znu* ABC and *znu* ABC2	MSΔΔ4 complemented with pMV306kan, containing genes *msmeg* _6045-47 and pMV306hyg, containing genes *msmeg* _6049-51	Goethe et al. 2021
MSΔΔ5::F_3	MSΔΔ5 complemented with *msp* D and own promoter	MSΔΔ5 complemented with pMV306hyg, containing *msmeg* _6057	this work
MSΔΔΔ7::F_3	MSΔΔΔ7 complemented with *msp* D and own promoter	MSΔΔ5 complemented with pMV306hyg, containing *msmeg* _6057	this work
MS::*msp* D-HA	MSwt with *in situ* HA-tagged *msp* D	MSwt carrying pKM461 transformed with pKM491 and Oligo oORBIT-MS_6057-HA	this work
MSΔ6::L	MSΔ6 complemented with HA-*zur*	MSΔ6 complemented with pMV306hyg containing HA-tagged *msmeg_* 4487 under control of the *msmeg_* 4486 promotor	Goethe et al. 2021

Plasmids

Abbreviation	Plasmids	Description	Origin
-	pMV306hyg/kan	integrative E.coli - mycobacterium shuttle vector, *hyg* **or** *kan*	Stover et al. 1991
-	pKM461	plasmid containing RecT recombinase, Bxb1 integrase and SacB for ORBIT mutagenisis	Murphy et al. 2018
-	pKM491	payload plasmid for C-terminal *in situ* FLAG-His-tagging of genes	Murphy et al. 2018
	pKM491-HA	payload plasmid for C-terminal *in situ* HA-tagging of genes	this work
-	p2NIL	*E. coli* - mycobacterium shuttle vector for gene manipulation, *kan*	Parish & Stoker 2000
-	pGOAL19	*hyg,* P_{AG85}-*lac* Z, P_{hsp60}-*sac* B, *Pac* I cassette vector, *amp*	Parish & Stoker 2000
	p2NIL-MS6057_AB	p2NIL containing 1500 bp up- and downstream fragments of *msmeg_* 6057	this work
	p2NIL-MS6057_Del	p2NIL containing *Pac* I restricted selection marker gene cassette of pGOAL19 and 1500 bp up- and downstream fragments of *msmeg* _6057	this work
F3	pMV306hyg-MS6057	pMV306hyg containing *msp* D (*msmeg* _6057) under its own promoter (NC_008596 pos. 6124345 - 6125368)	this work
A	pMV306kan-MSznuABC1	pMV306kan, containing genes msmeg_6045-47 with own promoter (NC_008596 pos. 6115073 - 6115257)	Goethe et al. 2021
B	pMV306hyg-MSznuABC2	pMV306kan, containing genes msmeg_6049-51 with own promoter (NC_008596 pos. 6120449 - 6120850)	Goethe et al. 2021
L	pMV306hyg-Mszur-HA	pMV306hyg containing HA-tagged Zur (*msmeg* _4487) under control of the *smt* B (*msmeg* _4486) promotor	Goethe et al. 2020

Goethe E, Laarmann K, Luhrs J, Jarek M, Meens J, Lewin A, Goethe R. 2020. Critical Role of Zur and SmtB in Zinc Homeostasis of Mycobacterium smegmatis. mSystems 5.

Goethe, E., et al., Identification and Characterization of Mycobacterium smegmatis and Mycobacterium avium subsp. paratuberculosis Zinc Transporters. Journal of bacteriology, 2021. 203(11): p. e00049–21.

Raleigh FA, Lech K, Brent R 1989: Selected topics from classical bacterial genetics. In Current protocols in molecular biology. Edited by Ausubel FM. New York, USA: Publishing Associates and Wiley Interscience:1.4.1-1.4.14.

Snapper SB, Melton RE, Mustafa S, Kieser T, Jacobs WR Jr. 1990. Isolation and characterization of efficient plasmid transformation mutants of Mycobacterium smegmatis. Mol.Microbiol. 4.11 : 1911-19.

Parish T, Stoker NG. 2000. Use of a flexible cassette method to generate a double unmarked Mycobacterium tuberculosis tlyA plcABC mutant by gene replacement. Microbiology 146 (Pt 8):1969-1975.

Stover CK, de lC, V, Fuerst TR, Burlein JE, Benson LA, Bennett LT 1991: New use of BCG for recombinant vaccines. Nature, 351: 456-460.

Murphy, K.C., et al., ORBIT: a new paradigm for genetic engineering of mycobacterial chromosomes. MBio, 2018. 9(6): p. 10.1128/mbio. 01467–18.

[a] The position numbers given related to GenBank accession no. NC_008596

Table T2: Oligonucleotides used in this study

No.	Oligonucleotides	Description or sequence (5’ to 3’)	Origin
1	oRT_MSMEG_3084fw (gapdh)	GTGCCAAGAAGGTCATCATC	Eckelt et al. 2014
2	oRT_MSMEG_3084rev (gapdh)	ACTCGTCATTGAGCACCTTG	Eckelt et al. 2014
3	oMSMEG_6057A_fw	GATCAAGCTTCCTCCCGAAAGGACTCTCAA	this work
4	oMSMEG_6057A_rev	GATCGAAGACGAGGTAGCGCACGATTGGGT	this work
5	oMSMEG_6057B_fw	GATCGAAGACGCTACCTGAACTAGGTCAAG	this work
6	oMSMEG_6057B_rev	CATGGTACCGGTGTGCGACGGTGAGGCGAT	this work
7	oMSMEG_6057Del_fw	CATCGCAGATCTCAATCAGC	this work
8	oMSMEG_6057Del_rev	GCCGCCGTATTGCACAATGC	this work
9	oRTMS6057_fw2	TGTTCGCTCTACTCGTGTCC	this work
10	oRTMS6057_rev2	GGAATGTCTCGGCTTGCT	this work
11	oProm-MspDfw	GATCTCTAGAGCCACGAGCTCTCGGATCGG	this work
12	oMS6057_rev	GTCAAGCTTCTAGTTCATGTTCCAGGGCTC	this work
13	opKM491-HA_fw	TGATGACTCGAGTCTAGAGCATGC	this work
14	opKM491-HA_rev	AGACTCGAGTCATCAAGCGTAATCTGGAACATCGTATGGGTA CTGGAAGTACAGGTTCTCGCCG	this work
15	oORBIT-MS_6057-HA	TCCGTTCGCCCGGTTGATCGCCTCGACGGGCGACAGCGTCA CCACCTACGGCGAGCCCTGGAACATGAACGGTTTGTCTGGT CAACCACCGCGGTCTCAGTGGTGTACGGTACAAACCTAGGT CAAGCGAATCGGCCGCCACGGGAATGCCGGCGGCCGACAT GGTGAACGCGCCGGTGGGCGGTACG	this work
16	oORBIT-oriE	CCTGGTATCTTTATAGTCCTGTCG	this work
17	oHAmspD_r	AACATCGTATGGGTAGTTCATGTTCCAGGG	this work
18	oRTmsmeg6047_fw	CTGGACGTTGTAGATGAGCA	Goethe et al. 2021
19	oRTmsmeg6047_rev	AATCGATGAAGCCGTACAAC	Goethe et al. 2021
20	oRTms6049_fw	CGGTGATTTCAAGGTGGAT	Goethe et al. 2021
21	oRTms6049_rev	ATCGATGACGTAGAGCTTGC	Goethe et al. 2021
22	oRTms6067_fw	GTGAAGCTGCGGTCCACT	this work
23	oRTms6067_rev	ACCGGATCGTATTTCCTGAG	this work
24	mspA-FP2	ATCGCGATGGTTGCAGCCA	Stephan et al. 2005
25	mspA-RP	GGTAGCCGAGTTCCAGCGT	Stephan et al. 2005

Eckelt E, Jarek M, Fromke C, Meens J, Goethe R. 2014. Identification of a lineage specific zinc responsive genomic island in Mycobacterium avium ssp. paratuberculosis. BMC Genomics 15:1076.

Goethe, E., et al., Identification and Characterization of Mycobacterium smegmatis and Mycobacterium avium subsp. paratuberculosis Zinc Transporters. Journal of bacteriology, 2021. 203(11): p. e00049–21.

File F1: Protein sequences of MspA and MspD without the signal sequence (marked red) used to create pore models using Swiss-Model Interactive Workspace (Waterhouse et al. 2018)

>MspD_WP_162139622.1
MFALLVSVTLVSPRPANAVDNQLSVVDGQGRTLTVQQAETFLNGVFPLDRNRLTREWFHSGRATYH
VAGPGADEFEGTLELGYQVGFPWSLGVGINFSYTTPNILIDGGDITQPPFGLDTIITPNLFPGVSISADL
GNGPGIQEVATFSVDVKGAKGAVAVSNAHGTVTGAAGGVLLRPFARLIASTGDSVTTYGEPWNMN

>MspA_WP_003891919.1
MKAISRVLIAMVAAIAALFTSTGTSHAGLDNELSLVDGQDRTLTVQQWDTFLNGVFPLDRNRLTREW
FHSGRAKYIVAGPGADEFEGTLELGYQIGFPWSLGVGINFSYTTPNILIDDGDITAPPFGLNSVITPNLF
PGVSISADLGNGPGIQEVATFSVDVSGPAGGVAVSNAHGTVTGAAGGVLLRPFARLIASTGDSVTTY
GEPWNMN

Waterhouse A, Bertoni M, Bienert S, Studer G, Tauriello G, Gumienny R, Heer FT, de Beer TAP, Rempfer C, Bordoli L, Lepore R, Schwede T. 2018. SWISS-MODEL: homology modelling of protein structures and complexes. NucAcRes 46, W1, W296-W303

Fig. S1: Expression of *msp* D in different mutant strains. MSwt (black bar), mutant strains lacking *msp* D alone (MSΔ3), *msp* D and *znu* ABC (MSΔΔ5) or *msp* D, *znu* ABC and *znu* ABC2 (MSΔΔΔ7) and *msp* D complemented strains (::F3) were grown in MBXT to an OD_{600} of 1.0. *msp* D expression was analyzed by qRT-PCR is are shown as rel. gene expression (log10), normalized to the housekeeping gene *gapdh* . N = 3 in duplicate.

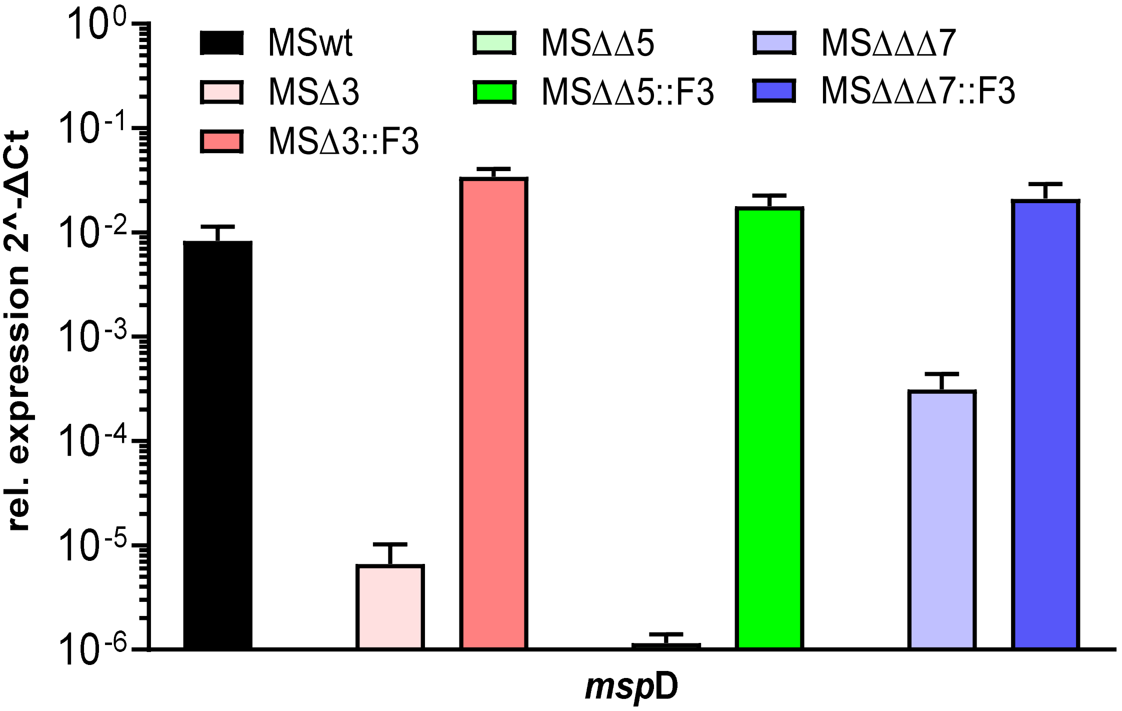

Fig. S2:Growth of different mutant strains in Sauton's medium. *Mycobacterium smegmatis* wildtype (MSwt, black) and mutant strains lacking *znu* ABC (MSΔ1, white) or *znu* ABC2 (MSΔ2, light green) and homologously complemented strains (MSΔ1::A, grey, MSΔ2::B, green) were precultured in MBXT and inoculated to Sauton`s medium with an OD_{600} of 0.1. Growth was monitored daily for 7 days by determination of OD_{600}. N = 3 in duplicate.

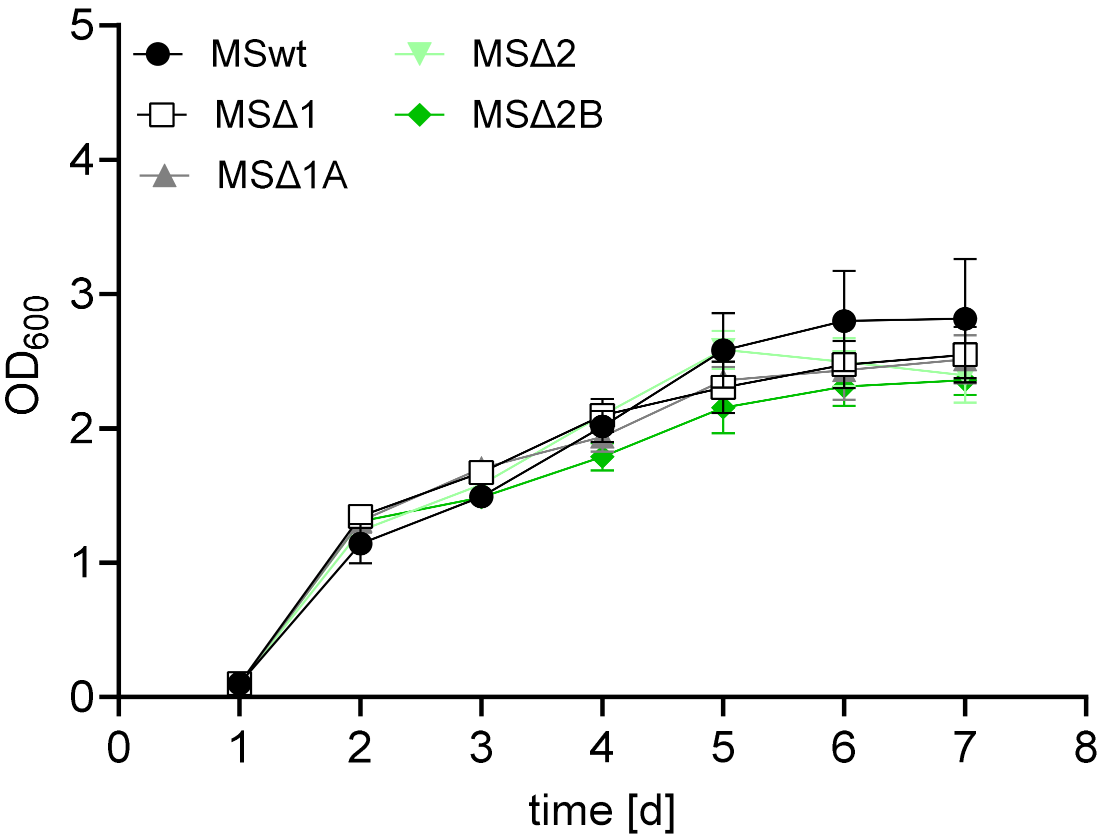

Fig. S3: Spot-On assays with different mutant strains. *Mycobacterium smegmatis* (MSMEG) wildtype (MSwt), MSMEG lacking *znu* ABC (MSΔ1), *znu* ABC2 (MSΔ2), *msp* D (MSΔ3), *msp* D and *znu* ABC (MSΔΔ5), and complemented strains (::A, ::B, ::F3) were grown in MBXT to an OD600 of 2-3, harvested, washed, inoculated in 1xPBS to an OD600 of 0.1, serially diluted up to 10*4 and spotted on 7H10 standard agar or on 7H10 supplemented with indicated substances. Plates were incubated for 4 days at 37°C. Shown are representative pictures of three independent experiments.

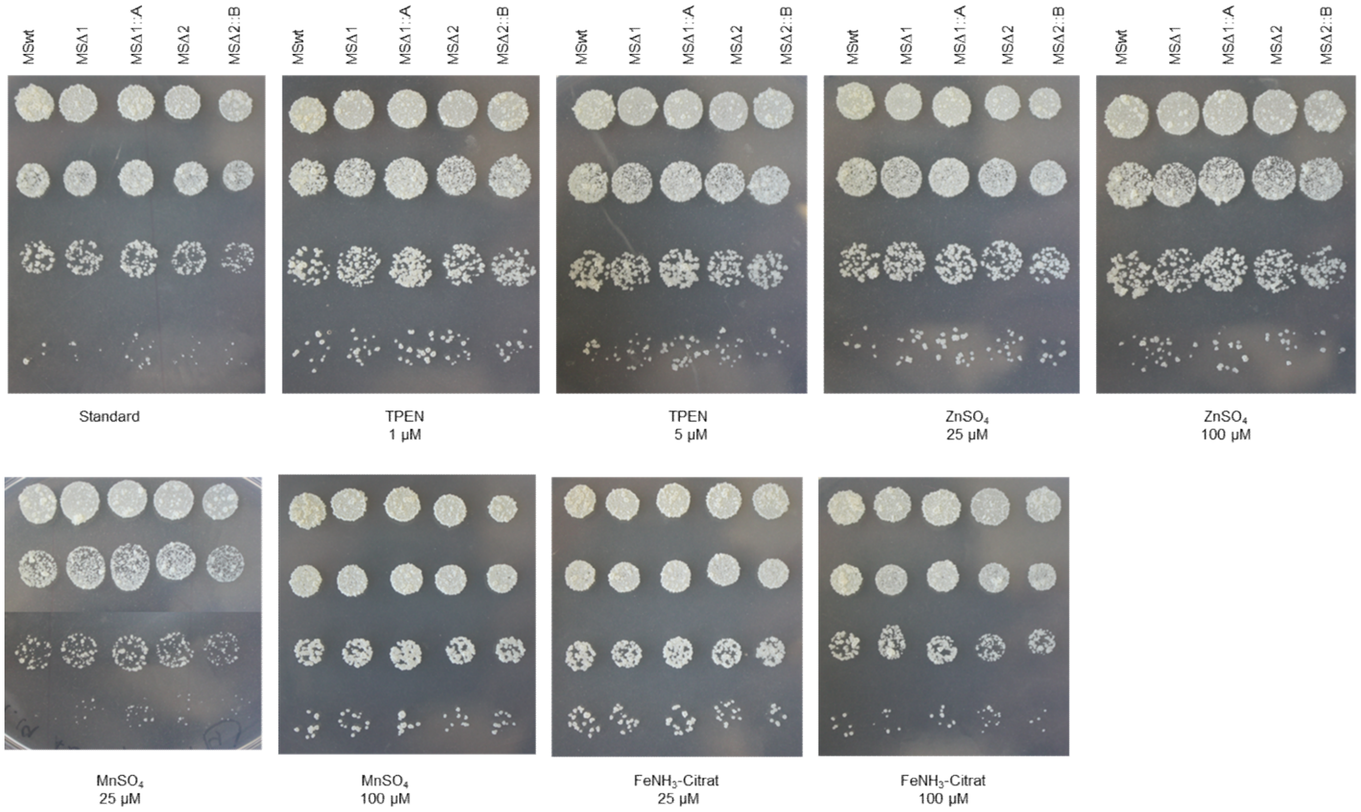

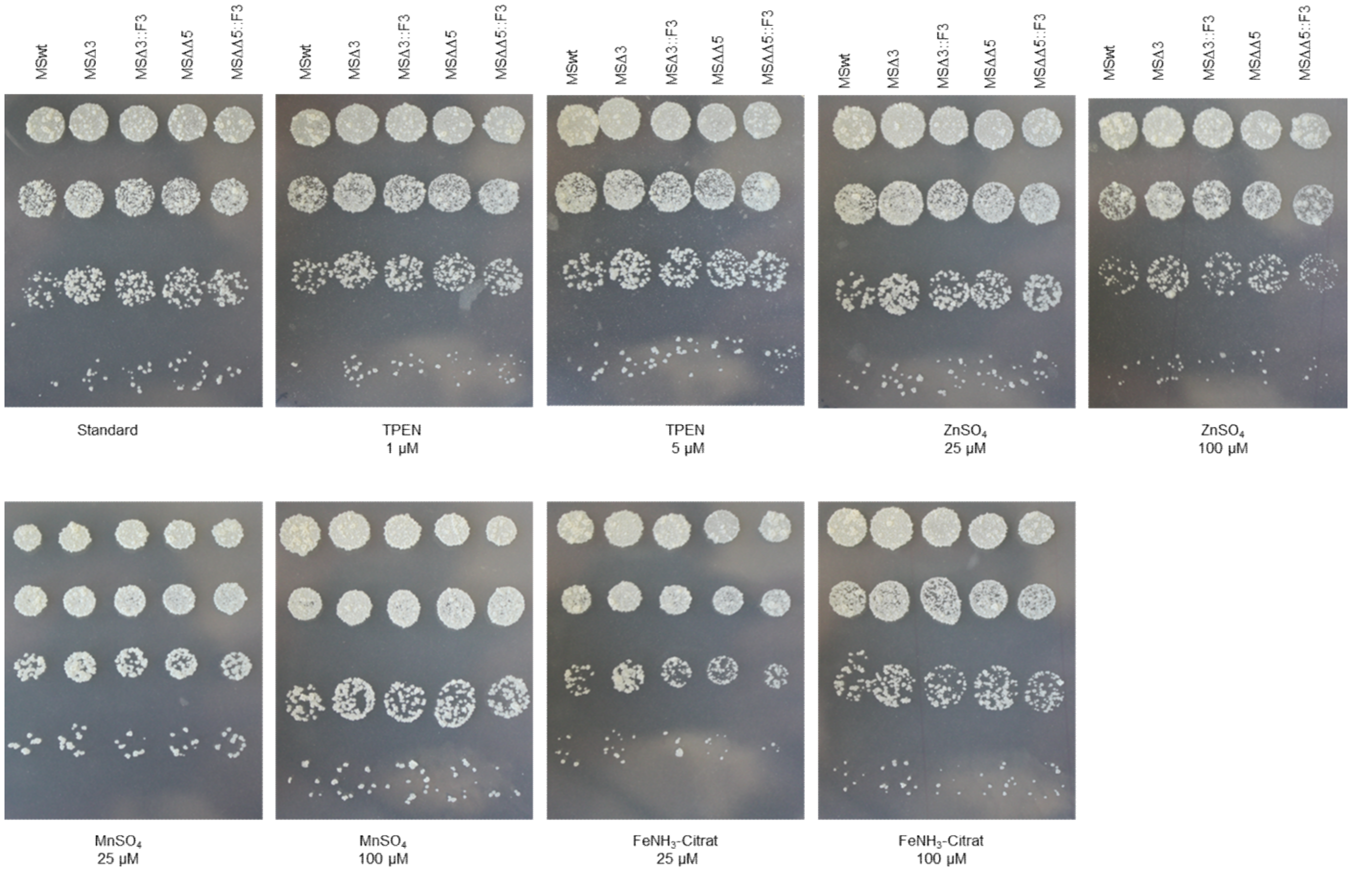
MSwt
MSΔ3
MSΔ3::F3
MSΔΔ5
MSΔΔ5::F3
Standard
TPEN 1 µM
TPEN 5 µM
ZnSO4 25 µM
ZnSO4 100 µM
MnSO4 25 µM
MnSO4 100 µM
FeNH3-Citrat 25 µM
FeNH3-Citrat 100 µM
